# Supplementary material for: Multi‐dataset identification of innovative feature genes and molecular mechanisms in keratoconus
Source: J Cell Mol Med. 2024 Sep 19;28(18):e70079. doi: 10.1111/jcmm.70079 (PMC11412914; doi:10.1111/jcmm.70079)
Supplement: Supplementary file 2 — Material S2. [file JCMM-28-e70079-s002.zip › 7.GSEA analysis/3.DBNDD1/KEGG_CELL_CYCLE.html]

Details for gene set KEGG\_CELL\_CYCLE[GSEA]

|  || Dataset | DBNDD1.group.cls#h\_versus\_l |
| Phenotype | group.cls#h\_versus\_l |
| Upregulated in class | l |
| GeneSet | KEGG\_CELL\_CYCLE |
| Enrichment Score (ES) | -0.42577803 |
| Normalized Enrichment Score (NES) | -1.4777149 |
| Nominal p-value | 0.13487476 |
| FDR q-value | 0.6075134 |
| FWER p-Value | 0.823 |
Table: GSEA Results Summary

  

Fig 1: Enrichment plot: KEGG\_CELL\_CYCLE      
 Profile of the Running ES Score & Positions of GeneSet Members on the Rank Ordered List

  

| SYMBOL | TITLE | RANK IN GENE LIST | RANK METRIC SCORE | RUNNING ES | CORE ENRICHMENT || 1 | CDC14A | na | 2027 | 0.298 | -0.0320 | No |
| 2 | CCNE2 | na | 4812 | 0.186 | -0.0958 | No |
| 3 | CDC14B | na | 6310 | 0.142 | -0.1264 | No |
| 4 | ANAPC1 | na | 6638 | 0.133 | -0.1240 | No |
| 5 | E2F2 | na | 6645 | 0.133 | -0.1123 | No |
| 6 | CCNA1 | na | 7073 | 0.123 | -0.1137 | No |
| 7 | SMAD2 | na | 7383 | 0.116 | -0.1122 | No |
| 8 | SKP2 | na | 8381 | 0.097 | -0.1324 | No |
| 9 | ESPL1 | na | 8518 | 0.094 | -0.1279 | No |
| 10 | PRKDC | na | 9157 | 0.083 | -0.1390 | No |
| 11 | CREBBP | na | 9242 | 0.082 | -0.1341 | No |
| 12 | CCND1 | na | 9676 | 0.076 | -0.1398 | No |
| 13 | MDM2 | na | 9756 | 0.075 | -0.1354 | No |
| 14 | RB1 | na | 10147 | 0.069 | -0.1405 | No |
| 15 | ATM | na | 11600 | 0.051 | -0.1779 | No |
| 16 | SMAD4 | na | 11961 | 0.047 | -0.1841 | No |
| 17 | ORC5 | na | 11992 | 0.047 | -0.1807 | No |
| 18 | SMC1B | na | 13223 | 0.036 | -0.2130 | No |
| 19 | RBX1 | na | 13500 | 0.034 | -0.2180 | No |
| 20 | TGFB3 | na | 13624 | 0.033 | -0.2186 | No |
| 21 | TP53 | na | 13962 | 0.030 | -0.2256 | No |
| 22 | FZR1 | na | 14075 | 0.029 | -0.2262 | No |
| 23 | HDAC1 | na | 14495 | 0.026 | -0.2360 | No |
| 24 | ANAPC4 | na | 14705 | 0.025 | -0.2398 | No |
| 25 | CDC23 | na | 14862 | 0.024 | -0.2421 | No |
| 26 | TFDP1 | na | 15222 | 0.021 | -0.2506 | No |
| 27 | ORC2 | na | 15315 | 0.020 | -0.2515 | No |
| 28 | CDC25A | na | 15496 | 0.019 | -0.2549 | No |
| 29 | ATR | na | 15758 | 0.017 | -0.2609 | No |
| 30 | STAG2 | na | 15784 | 0.017 | -0.2601 | No |
| 31 | ANAPC11 | na | 16026 | 0.016 | -0.2657 | No |
| 32 | BUB3 | na | 16101 | 0.015 | -0.2665 | No |
| 33 | PCNA | na | 16348 | 0.014 | -0.2724 | No |
| 34 | ORC4 | na | 16432 | 0.013 | -0.2736 | No |
| 35 | YWHAB | na | 16820 | 0.011 | -0.2838 | No |
| 36 | ANAPC5 | na | 17097 | 0.009 | -0.2910 | No |
| 37 | CCNE1 | na | 17317 | 0.007 | -0.2967 | No |
| 38 | ANAPC7 | na | 17646 | 0.005 | -0.3057 | No |
| 39 | CUL1 | na | 17786 | 0.004 | -0.3093 | No |
| 40 | CCND2 | na | 17929 | 0.003 | -0.3131 | No |
| 41 | STAG1 | na | 18413 | -0.000 | -0.3271 | No |
| 42 | ANAPC13 | na | 18434 | -0.000 | -0.3276 | No |
| 43 | CDKN2D | na | 18973 | -0.004 | -0.3428 | No |
| 44 | CCNH | na | 19151 | -0.006 | -0.3474 | No |
| 45 | SKP1 | na | 19198 | -0.006 | -0.3482 | No |
| 46 | TGFB2 | na | 19303 | -0.007 | -0.3506 | No |
| 47 | YWHAZ | na | 19676 | -0.010 | -0.3604 | No |
| 48 | ANAPC10 | na | 19847 | -0.012 | -0.3643 | No |
| 49 | PTTG2 | na | 19926 | -0.012 | -0.3654 | No |
| 50 | MAD1L1 | na | 19943 | -0.012 | -0.3648 | No |
| 51 | SMAD3 | na | 19999 | -0.013 | -0.3652 | No |
| 52 | CDC16 | na | 20107 | -0.014 | -0.3671 | No |
| 53 | SMC1A | na | 20200 | -0.015 | -0.3684 | No |
| 54 | PTTG1 | na | 20284 | -0.015 | -0.3694 | No |
| 55 | SFN | na | 21113 | -0.023 | -0.3913 | No |
| 56 | CDK4 | na | 21131 | -0.023 | -0.3897 | No |
| 57 | CDKN2A | na | 21295 | -0.025 | -0.3922 | No |
| 58 | ORC3 | na | 21357 | -0.025 | -0.3917 | No |
| 59 | ABL1 | na | 21453 | -0.026 | -0.3921 | No |
| 60 | CDKN2C | na | 21480 | -0.027 | -0.3905 | No |
| 61 | CCNB3 | na | 21737 | -0.029 | -0.3952 | No |
| 62 | YWHAG | na | 22158 | -0.034 | -0.4044 | No |
| 63 | CDC26 | na | 22265 | -0.035 | -0.4043 | No |
| 64 | YWHAE | na | 22319 | -0.035 | -0.4027 | No |
| 65 | CCND3 | na | 22320 | -0.035 | -0.3996 | No |
| 66 | MAD2L1 | na | 22334 | -0.035 | -0.3968 | No |
| 67 | RAD21 | na | 22447 | -0.037 | -0.3967 | No |
| 68 | GSK3B | na | 22452 | -0.037 | -0.3936 | No |
| 69 | CDC25B | na | 22622 | -0.039 | -0.3950 | No |
| 70 | RBL2 | na | 22650 | -0.039 | -0.3923 | No |
| 71 | MYC | na | 22660 | -0.039 | -0.3891 | No |
| 72 | ANAPC2 | na | 22849 | -0.041 | -0.3908 | No |
| 73 | CDKN1B | na | 22949 | -0.042 | -0.3899 | No |
| 74 | YWHAQ | na | 23046 | -0.044 | -0.3888 | No |
| 75 | CDC27 | na | 23312 | -0.047 | -0.3922 | No |
| 76 | E2F3 | na | 24031 | -0.057 | -0.4079 | No |
| 77 | HDAC2 | na | 24190 | -0.059 | -0.4072 | No |
| 78 | EP300 | na | 24292 | -0.060 | -0.4048 | No |
| 79 | SMC3 | na | 24731 | -0.067 | -0.4115 | No |
| 80 | MCM3 | na | 24829 | -0.068 | -0.4082 | No |
| 81 | DBF4 | na | 24955 | -0.070 | -0.4055 | No |
| 82 | MAD2L2 | na | 25657 | -0.081 | -0.4185 | Yes |
| 83 | E2F4 | na | 25756 | -0.083 | -0.4140 | Yes |
| 84 | TFDP2 | na | 25914 | -0.086 | -0.4108 | Yes |
| 85 | CDK1 | na | 25994 | -0.087 | -0.4053 | Yes |
| 86 | GADD45G | na | 26045 | -0.088 | -0.3989 | Yes |
| 87 | CDC20 | na | 26050 | -0.089 | -0.3911 | Yes |
| 88 | CDK7 | na | 26230 | -0.092 | -0.3880 | Yes |
| 89 | CDKN2B | na | 26262 | -0.093 | -0.3806 | Yes |
| 90 | MCM7 | na | 26354 | -0.095 | -0.3748 | Yes |
| 91 | CCNB1 | na | 26847 | -0.104 | -0.3797 | Yes |
| 92 | E2F5 | na | 26930 | -0.106 | -0.3726 | Yes |
| 93 | PKMYT1 | na | 27115 | -0.110 | -0.3680 | Yes |
| 94 | CDK6 | na | 27414 | -0.117 | -0.3662 | Yes |
| 95 | MCM4 | na | 27439 | -0.117 | -0.3564 | Yes |
| 96 | CCNB2 | na | 27443 | -0.118 | -0.3460 | Yes |
| 97 | CHEK2 | na | 27531 | -0.119 | -0.3378 | Yes |
| 98 | E2F1 | na | 27800 | -0.126 | -0.3343 | Yes |
| 99 | WEE1 | na | 27928 | -0.129 | -0.3264 | Yes |
| 100 | CDC7 | na | 28134 | -0.134 | -0.3204 | Yes |
| 101 | MCM2 | na | 28139 | -0.134 | -0.3085 | Yes |
| 102 | MCM5 | na | 28262 | -0.137 | -0.2998 | Yes |
| 103 | ZBTB17 | na | 28294 | -0.138 | -0.2884 | Yes |
| 104 | CDK2 | na | 28335 | -0.139 | -0.2771 | Yes |
| 105 | GADD45A | na | 28540 | -0.145 | -0.2701 | Yes |
| 106 | RBL1 | na | 29030 | -0.158 | -0.2701 | Yes |
| 107 | WEE2 | na | 29588 | -0.175 | -0.2706 | Yes |
| 108 | YWHAH | na | 29598 | -0.176 | -0.2551 | Yes |
| 109 | MCM6 | na | 29866 | -0.184 | -0.2464 | Yes |
| 110 | ORC6 | na | 30193 | -0.194 | -0.2385 | Yes |
| 111 | CDKN1C | na | 30428 | -0.201 | -0.2272 | Yes |
| 112 | TTK | na | 30521 | -0.204 | -0.2116 | Yes |
| 113 | GADD45B | na | 30544 | -0.205 | -0.1940 | Yes |
| 114 | CCNA2 | na | 30766 | -0.213 | -0.1813 | Yes |
| 115 | TGFB1 | na | 30914 | -0.218 | -0.1661 | Yes |
| 116 | CHEK1 | na | 31495 | -0.240 | -0.1615 | Yes |
| 117 | CDKN1A | na | 31724 | -0.250 | -0.1457 | Yes |
| 118 | BUB1 | na | 31777 | -0.253 | -0.1247 | Yes |
| 119 | CDC45 | na | 31848 | -0.256 | -0.1038 | Yes |
| 120 | CDC25C | na | 32054 | -0.267 | -0.0859 | Yes |
| 121 | BUB1B | na | 33216 | -0.346 | -0.0886 | Yes |
| 122 | ORC1 | na | 33535 | -0.373 | -0.0645 | Yes |
| 123 | PLK1 | na | 34377 | -0.506 | -0.0436 | Yes |
| 124 | CDC6 | na | 34577 | -0.602 | 0.0044 | Yes |
Table: GSEA details [plain text format]

  

Fig 2: KEGG\_CELL\_CYCLE      
 Blue-Pink O' Gram in the Space of the Analyzed GeneSet

  

Fig 3: KEGG\_CELL\_CYCLE: Random ES distribution      
 Gene set null distribution of ES for **KEGG\_CELL\_CYCLE**
